# RNAi-Mediated Gene Suppression in a GCAP1(L151F) Cone-Rod Dystrophy Mouse Model

**DOI:** 10.1371/journal.pone.0057676

**Published:** 2013-03-05

**Authors:** Li Jiang, Tansy Z. Li, Shannon E. Boye, William W. Hauswirth, Jeanne M. Frederick, Wolfgang Baehr

**Affiliations:** 1 Department of Ophthalmology and Visual Sciences, University of Utah Health Science Center, Salt Lake City, Utah, United States of America; 2 Department of Ophthalmology, University of Florida College of Medicine, Gainesville, Florida, United States of America; 3 Department of Biology, University of Utah, Salt Lake City, Utah, United States of America; 4 Department of Neurobiology and Anatomy, University of Utah Health Science Center, Salt Lake City Utah, United States of America; National Eye Institute, United States of America

## Abstract

Dominant mutations occurring in the high-affinity Ca^2+^-binding sites (EF-hands) of the *GUCA1A* gene encoding guanylate cyclase-activating protein 1 (GCAP1) cause slowly progressing cone-rod dystrophy (CORD) in a dozen families worldwide. We developed a nonallele-specific adeno-associated virus (AAV)-based RNAi knockdown strategy to rescue the retina degeneration caused by GCAP1 mutations. We generated three genomic transgenic mouse lines expressing wildtype (WT) and L151F mutant mouse GCAP1 with or without a C-terminal GFP fusion. Under control of endogenous regulatory elements, the transgenes were expressed specifically in mouse photoreceptors. GCAP1(L151F) and GCAP1(L151F)-GFP transgenic mice presented with a late onset and slowly progressive photoreceptor degeneration, similar to that observed in human GCAP1-CORD patients. Transgenic expression of WT GCAP1-EGFP in photoreceptors had no adverse effect. Toward therapy development, a highly effective anti-mGCAP1 shRNA, mG1hp4, was selected from four candidate shRNAs using an *in-vitro* screening assay. Subsequently a self-complementary (sc) AAV serotype 2/8 expressing mG1hp4 was delivered subretinally to GCAP1(L151F)-GFP transgenic mice. Knockdown of the GCAP1(L151F)-GFP transgene product was visualized by fluorescence live imaging in the scAAV2/8-mG1hp4-treated retinas. Concomitant with the mutant GCAP1-GFP fusion protein, endogenous GCAP1 decreased as well in treated retinas. We propose nonallele-specific RNAi knockdown of GCAP1 as a general therapeutic strategy to rescue any GCAP1-based dominant cone-rod dystrophy in human patients.

## Introduction

Cone-rod dystrophy (CORD, with a prevalence of 1/40,000) is a rare, highly heterogeneous class of hereditary retinal disease inherited in a dominant, recessive or X-linked fashion [1]. The disease manifests with photoaversion, reduced central visual acuity, achromatopsia at early stages, and eventually loss of peripheral vision attributed to progressive loss of first cone and then rod photoreceptors. Thus far, 27 genes have been linked to cone-rod dystrophy; of these, ten genes are associated with dominant CORD, 15 with recessive CORD, two are X-linked (RetNet, https://sph.uth.tmc.edu/retnet/). The protein products of these genes are involved in multiple aspects of photoreceptor structure and function [2]. One of the best characterized dominant CORD genes is *GUCA1A* encoding guanylate cyclase-activating protein 1 (GCAP1 [3]. Worldwide, about one dozen families with more than 100 affected members have been identified to date [4].

GCAP1 plays a key role in accelerating guanylate cyclase activity in retinal photoreceptors. Rod phototransduction is regulated by two guanylate cyclases (GC1 and GC2) and two GCAPs (GCAP1 and GCAP2) [5]. The two GCAP genes (*Guca1a* and *Guca1b*) are arranged in a tail-to-tail array on mouse chromosome 17 [6] while the GC genes (*Gucy2e* and *Gucy2f*) are located on different chromosomes (chromosomes 11 and X, [7]). The two GCAPs overlap partially in regulating the GCs of rods, and both contribute to rod recovery after photolysis [8]–[11]. In cones, only GCAP1 is involved in regulating GC1, the predominant GC of cone phototransduction. Germline deletion of both GCAPs renders GC activity Ca^2+^ insensitive; flash responses from dark-adapted rods were larger and slower, and recovery to the dark state was delayed [8]. By contrast, cone ERGs recorded from GCAPs^−/−^ mice had normally saturated a-wave and b-wave amplitudes and increased sensitivity of both M- and S-cone systems [12]. Both the cone driven b-wave and a-wave were delayed similarly as observed in rods. Transgenic GCAP1 could restore normal rod and cone response recovery [9], [12].

**Figure 1:**
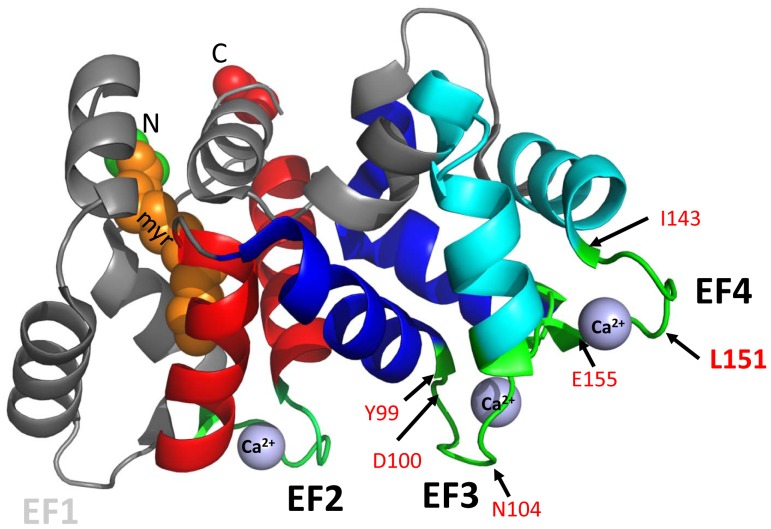
GCAP1 mutations causative for CORD. Ribbon structure of N-myristoylated GCAP1. N, N-terminus, C, C-terminus; myr, myristoyl side chain (orange) attached to Gly-2; Ca^2+^ ions (blue-gray). EF-hands consist of helix-loop-helix domains. Two helices flanking EF-hand 2 (EF2, red), two helices flanking EF3 (dark blue) and the two helices flanking EF4 (light blue) are shown. Amino acid residues mutated in dominant CORD cluster around EF3 and EF4. Positions (black arrows) of mutant residues (red) are indicated: E89K, Y99C, D100E and N104K are located in EF-hand 3 (EF3), while I143NT, L151F and E155G are located in EF-hand 4 (EF4).

As members of the calmodulin superfamily, GCAPs feature four EF-hand motifs (EF1-4) for Ca^2+^-binding [3], [13]. Three of these (EF2-4) have been established as high-affinity Ca^2+^-binding sites, whereas motif EF1 is incompatible with Ca^2+^-binding as key residues essential for Ca^2+^ coordination are absent [14]. Mutations in GCAP1, but not GCAP2, have been associated with autosomal dominant CORD3 [15]. Missense mutations in GCAP1 include EF3 mutations (E89K, Y99C, D100E, N104K) and EF4 mutations (I143NT, **L151F**, E155G, G159V) (**Fig. 1**) [16]–[23]. These dominant GCAP1 mutations alter Ca^2+^-association, decrease Ca^2+^ sensitivity and produce constitutive activity of photoreceptor guanylate cyclase 1 at normal dark Ca^2+^ levels, and persistent stimulation of GC1 in the dark [21], [22]. Elevated intracellular cGMP and Ca^2+^ trigger cell death which can be ameliorated by increased cGMP hydrolysis [24].

The L151F mutation was identified in two independent families with cone and cone-rod dystrophy, respectively [17], [23]. In the first family [17], dyschromatopsia, hemeralopia and reduced visual acuity were evident by the second-to-third decades of life and photopic electroretinographic (ERG) responses were non-recordable. In the second, five-generation family [23], 11 of 24 individuals were affected by photoaversion, color vision defects, central acuity loss and legal blindness in decades 2–3 of life. The causative GCAP1(L151F) mutation, a conservative substitution, disrupted Ca^2+^ coordination at EF-hand 4 and changed Ca^2+^ sensitivity. Molecular dynamics suggested a significant decrease in Ca^2+^-binding to EF-hand motifs 2 and 4, with an overall shape change of the L151F-GCAP1 molecule relative to wildtype [17].

Two mouse models of retinal dystrophy associated with GCAP1 mutations have been generated previously. The first, a transgenic mouse model expressing bovine GCAP1(Y99C) under control of the opsin promoter, produced severe and rapid rod-cone degeneration resembling retinitis pigmentosa [25]. The second model, a GCAP1(E155G) knock-in mouse, displayed late-onset and slowly progressive cone-rod photoreceptor degeneration, mimicking human CORD [26]. In this study, we generated three transgenic mouse lines expressing wildtype and L151F mutant GCAP1 with or without a C-terminal EGFP fusion. The EGFP tag was instrumental toward developing an effective knockdown RNA interference and served to distinguish transgenic GCAPs from endogenous GCAPs. All transgenes contained the complete mouse *Guca1a* gene, including native regulatory elements. Mutant transgenic mice develop retina pathology slowly and recapitulate features of human CORD. To develop a vector suitable for knock-down gene therapy, we generated scAAV2/8 virus that expresses a nonallele-specific shRNA that targets both mutant and native *Guca1a* mRNAs. By immunoblot and *in-vivo* fundoscopy, we show that both transgenic and endogenous GCAP1 were down-regulated effectively in AAV-treated retinas. These data establish shRNA-mediated RNAi as a potential therapeutic strategy for adCORD patients carrying any EF-hand *GUCA1A* mutation.

## Materials and Methods

### Mice (Ethics Statement)

Procedures for the animal experiments of this study were IACUC-approved by the University of Utah and conformed to recommendations of the Association of Research for Vision and Ophthalmology (ARVO). Transgenic and wildtype (WT) mice were maintained under 12-hour cyclic dark/light conditions.

### Cloning of mGCAP1 Genomic Constructs

A 14,832 bp mouse GCAP1 genomic sequence (mG1) was modified to generate three transgenes which expressed either wildtype GCAP1 fused to EGFP (G1-GFP), or one of two mutant proteins, G1(L151F) and G1(L151F)-GFP. To generate a GFP fusion mGCAP1 transgenic construct, G1-GFP**,** a chloramphenicol-resistant cassette flanked by AsisI and AscI sites was first inserted into the wildtype mGCAP1 genomic construct right before the stop codon using a homologous recombination method, termed ET cloning [27]. We amplified by PCR the AsisI-AscI chloramphenicol-resistant cassette with primer pair, G1_CmRasisIF: 5′-GCGAACACGAGGAGGCAGGCACCGGCGACCTGGCAGCGGAGGCTGCGGGTGCGATCGCagcattacacgtcttgagcgattgt, and G1_CmRascIR: 5′-ACCGCACGGGGCCAGCCCTCAGCAGGCAGAAGCCACAGGGTGAATGC**TCA** GGCGCGCCCacttaacggctgacatgggaatta. Relevant to primer design are regions homologous to 5′ and 3′ flanking sequences (50-bp, shown in black) of the mGCAP1 stop codon (bold-faced) in the transgene, AsisI and AscI restriction sites (underlined), and regions complementary to the 5′ and 3′ sequences of a chloramphenicol-resistant cassette (lower case), respectively. The purified PCR product of the chloramphenicol-resistant cassette was co-electroporated with the mGCAP1 transgenic construct (containing an ampicillin-resistant cassette) into competent cells containing an inducible Red recombinase. Ampicillin and chloramphenicol ‘double’-resistant colonies were selected, in which a Red homologous recombination occurred and indicated that the chloramphenicol-resistant cassette with flanking AsisI and AscI sites was inserted immediately before the mGCAP1 stop codon. Subsequently, we replaced the chloramphenicol-resistant cassette in the recombinant mGCAP1 genomic construct with an AsisI-AscI EGFP cassette.

To generate the G1 (L151F) mutant transgene, a C to T point mutation in codon 151 was introduced by site-directed mutagenesis into the AatII-AatII mGCAP1 fragment (3.3-kb) containing codon L151 (Stratagene, La Jolla, CA). The pair of DNA oligonucleotides used for mutagenesis was: G1(L151F)_F: 5′-TTTCTCTCCATCCCAGGGGAATTGTCCCTGGAGGAG, and G1(L151F)_R: 5′- CATGAACTCCTCCAGGGACAATTCCCCTGGGATGGA. The mutated AatII-AatII fragment was then substituted for the wild-type counterpart in the mG1 genomic construct. The GFP fusion mutant transgene, G1(L151F)-GFP, was generated by replacing a 2-kb SgrAI-ClaI fragment in G1 (L151F) with a 2.8-kb SgrAI-ClaI fragment containing GFP from G1-GFP. All transgenes were confirmed by DNA sequencing within the pBSKS (+) vector.

### Generation of Genomic Transgenic Mice

The three transgenes were released from pBSKS (+) vector by NotI digestion and microinjected into the pronuclei of fertilized mouse FVB/N oocytes to produce transgenic mice at the University of Utah core facility. Transgenic mice were identified by PCR genotyping with primers **G1T_F:** 5′-ATAGGGCGTCGACTCGATCACGCAGC, and **G1T_R:**5′- TAAGGGCGGAAGATCACGGAGGTAGC **)**. The primers are located across the 3′ boundary of the transgene and the pBSKS (+) backbone. A diagnostic fragment of 540-bp discriminates the transgenes from the endogenous GCAP1 gene. The C to T point mutation (L151F) was confirmed by DNA sequencing in the G1(L151F) and G1(L151F)-GFP transgenic founder mice. Transgenic mice were outbred to C57BL/J mice.

### Western Blotting

Cultured cells and mouse retinas were lysed by sonication in RIPA buffer (150 mmol/l NaCl, 1% NP-40, 0.5% sodium deoxycholate, 1% SDS, 50 mmol/l Tris pH 8.0). The supernatant of each lysate was separated on a 10% SDS-PAGE(∼15 µg protein/well), and then transferred to a nitrocellulose membrane (Biorad, Hercules, CA). Subsequently, the membrane was probed with primary antibodies (anti-GCAP1 polyclonal antibody, UW101, 1∶5,000 or anti-β actin monoclonal antibody, 1∶3,000) followed by HRP-conjugated secondary antibody. Phosphorescence (ECL system, NEN Life Science, Boston, MA) was used to visualize the signal on X-ray film.

### Immunocytochemistry

Mouse eyes were dissected and immediately immersion-fixed with 4% paraformaldehyde in 0.1 M phosphate buffer, pH 7.4, for 2 hours on ice. After removal of the anterior segment, the eyecups were equilibrated sequentially with 15% and 30% sucrose in phosphate buffer for cryoprotection, and then embedded in OCT. Subsequently, retina cryosections of 12 µm thickness were cut and used for direct fluorescence confocal microscopy or immunohistochemical analysis. For immunohistochemistry, retina sections were incubated with primary antibody (UW101, 1∶2,000, 4°C) overnight, followed by fluorescence-conjugated secondary antibody (1∶300, 21°C) for 1 hour in a humidified chamber. Sections were washed with phosphate buffer for 5 minutes (X3) between incubations. To label nuclei, 4′,6-diamidino-2-phenylindole (DAPI, 1∶5,000) was added to the solution containing secondary antibody; retina sections were incubated with DAPI for one hour if imaging by direct fluorescence microscopy. After applying a drop of anti-fade agent, the sections were imaged with an Olympus Fluoview (model FV 1000) inverted confocal microscope.

### Histology

Mouse eyeballs were fixed by immersion in 2% glutaraldehyde-1% paraformaldehyde in 0.1 M cacodylate, pH 7.4, overnight at 4°C. Following removal of the anterior segment, the eyecups were postfixed in 1% osmium oxide in the same buffer for 1 hour, dehydrated with an ascending series of ethanols, infiltrated with ethanol:plastic mixtures and embedded in Spurr’s resin (Ted Pella, Inc., Redding, CA). Retina sections (1 µm) passing through the optic nerve were cut with an ultramicrotome and contrasted with Richardson’s stain. Bright-field images of retina histology were acquired using a Zeiss Axiovert 200 (Carl Zeiss Inc., Thornwood, NY) microscope and 62x objective. Images across entire retina sections were acquired with a 20x objective and imported into image J software with scaling of 3.8 pixels/µm. Outer nuclear layer (ONL) thicknesses were measured at 250 µm intervals from the optic nerve head.

### Electroretinography (ERG)

Scotopic and photopic ERG responses of control and transgenic mice were recorded as described [4]. Mice were dark-adapted overnight, anesthetized by intraperitoneal injection of 10 mg/ml ketamine-1 mg/ml xylazine (10 µl/g body weight) in PBS under dim red illumination, and kept comfortable on a heating pad. After dilating pupils by applying a drop of 2.5% phenylephrine (Akorn, Inc., Decatur, IL), ERG responses were recorded from 3–5 mice of each genotype per time point using a UTAS E-3000 system (LKC Technologies,Inc., Gaithersburg, MD). For scotopic ERG, mice were tested at intensities ranging from −40 decibels (db) (−3.4 logcds m^–2^) to 25 db (2.9 log cds m^–2^) without initial rod saturation. For photopic ERG, a rod saturating background light of 10 db (1.48 log cds m^–2^) was applied for 20 minutes before and during recording. Single flash responses were usually recorded at stimulus intensities of −10 db (−0.6 log cds m^–2^) to 25 db (2.9 log cds m^–2^). Five or fewer flashes were averaged per intensity level, with longer flash intervals with increasing intensity. Triple-antibiotic ointment was routinely placed on the eye to prevent infection after ERG testing.

### OptoMotry

Transgenic and control mice were tested for spatial visual acuity using an Optomotry apparatus which facilitates rapid screening of functional vision [28]. Briefly, mice were placed singly on a platform centered within a quad-square formed by four inward-facing computer screens, and observed by an overhead video camera. Vertical sine-wave gratings were presented at photopic luminance levels on screens rotating 12 deg/sec (speed) either clockwise or counterclockwise, as determined randomly by OptoMotry© software. The mouse tracks the grating with reflexive head movements when the rotating grating is perceptible. By observing mouse tracking, spatial frequency thresholds were quantified by increasing the spatial frequency of the rotating grating (usually between 0.03–0.35 grating cycles/degree) until finding a maximum frequency at which the mice track.

### Constructs Expressing Mouse GCAP1-EGFP and GCAP1_shRNA*in vitro*

Using shRNA prediction tools provided *online* by Whitehead, Ambion, Invitrogen and Dharmacon, we designed four putative shRNAs (mG1hp1-4) specifically targeting mouse GCAP1 and a mismatch control (mG1hp4m2) carrying the central two-nucleotide mutation in the mG1hp4 guide strand (GG to CC). To avoid possible off-target effects, an NCBI BLAST homology search was performed for the candidate shRNAs and no significant homology with other mouse genes or sequences was identified. A short-hairpin RNA (shRNA) expression vector, pmC_hH1, containing a shRNA expression promoter (human H1 promoter, hH1) and a CMV promoter-driven mCherry reporter, was used to generate anti-mGCAP1 shRNA constructs expressing mG1hp1-4 and mG1hp4m2 [4]. The construct mG1-EGFP, expressing EGFP fusion mouse GCAP1, was generated by cloning the mouse GCAP1 cDNA into pEGFP-N1 vector (Clontech, Mountain View, CA) downstream of the CMV promoter.

### Knockdown Efficiency Assay in Cell Culture

HEK293 cells (ATCC, CRL-1573) were cultured in DMEM supplemented with 10% fetal calf serum and 100 u/ml of penicillin-streptomycin (Invitrogen, Carlsbad, CA). In 12-well plates, HEK293 cells were cotransfected with mG1-EGFP plasmids and anti-mGCAP1 shRNA expression plasmids, mG1hp1-4 or mG1hp4m2, at different mass ratios (1∶3–3∶1) with a total amount of 1.5 µg/well (normalized by pBS vector DNA). At 48 hours post-transfection, expression of mG1-GFP and mCherry reporter in the transfected HEK293 cells was directly analyzed by live cell fluorescence microscopy using a Zeiss Axiovert 200 microscope. At 50 hours post-transfection, the cotransfected cells were harvested to analyze protein levels of mG1-GFP by immunoblotting; β-actin was used as an endogenous control.

### Preparation of scAAV2/8 Virus Expressing Anti-mGCAP1 shRNA

Self-complimentary AAV2/8 (scAAV2/8) was used to express mG1hp4 and its mismatched control, mG1hp4m2, in the mouse retina. The scAAV2/8 packaging constructs were generated by cloning hH1 promoter-driven mG1hp4 and mG1hp4m2 cassettes into a shuttle vector pscAAV-CAG-mCherry, which was modified from pscAAV-CAG-hGFP (provided by W.W. Hauswirth). The two plasmid cotransfection method was used to produce scAAV2/8 viral particles expressing mG1hp4/4m2 in collaboration with the University of Florida [29]. Vector genome-containing viral particles were titered by real-time PCR and resuspended in a balanced salt solution (Alcon Laboratories, Fort Worth, TX) containing 0.014% Tween-20 at a concentration of ∼1.0 ×10^12^vector genomes per milliliter (vg/ml).

### Mouse Subretinal Injection

G1(L151F)-GFP mice were injected subretinally with scAAV2/8 on postnatal day 30 (P30) as described [4]. Briefly, after anesthesia with a ketamine-xylazine solution (see ERG protocol), 0.5% proparacaine solution (Alcon Laboratories Inc., Fort Worth, TX) was applied to the cornea as a topical anesthetic. A small puncture hole was made through the cornea with a 30½ -gauge beveled needle (Becton Dickinson & Company, Franklin Lakes, NJ). A 33-gauge blunt needle attached to a microliter syringe (Hamilton Bonaduz, Switzerland) was introduced tangentially through the hole, and 1 µl of scAAV2/8 virus was delivered subretinally. Following injection, a triple-antibiotic ointment containing bacitracin, neomycin sulfate and polymyxin B (Taro Pharmaceuticals, Inc., Hawthorne, NY) was placed on the eye to prevent infection. Injections were performed in left eyes only, leaving the right eyes as untreated controls.

### *In-vivo* Fluorescence Imaging of the Mouse Retina

Fluorescence live imaging of mouse retinas was used to detect the expression of GFP fusion GCAP1 transgenes, G1-GFP and G1(L151F)-GFP, in mice 1–2 months of age, as well as in the knockdown of mG1(L151F)-GFP in transgenic mice one-month posttreatment with scAAV2/8. Mice were anesthetized with isoflurane gas (2%–3%) and their pupils were dilated with 2.5% phenylephrine (Akorn, Inc., Decatur, IL). After applying 0.5% proparacaine solution (Alcon Laboratories Inc., Fort Worth, TX) to the cornea as topical anesthetic, a glass coverslip was placed on the mouse eye to maintain corneal moisture and reduce refractive error. The mouse was stabilized during imaging on a custom-designed heated aluminum stage specifically fit for the microscope (Zeiss LSM 700; Carl Zeiss, Inc., Thornwood, NY). Fluorescent fundus images were acquired with a 5x air objective and Zeiss image acquisition software. Triple-antibiotic ointment was placed on the eye to prevent infection after imaging.

### Statistical Data Analysis

ONL thicknesses and the ERG response amplitudes were analyzed by two-way ANOVA, while the Optomotry data was analyzed by single-way ANOVA. The level of statistical significance was set at p = 0.05.

## Results

### Generation of Transgenic Mice and Expression of Mutant GCAP1

**Figure 2:**
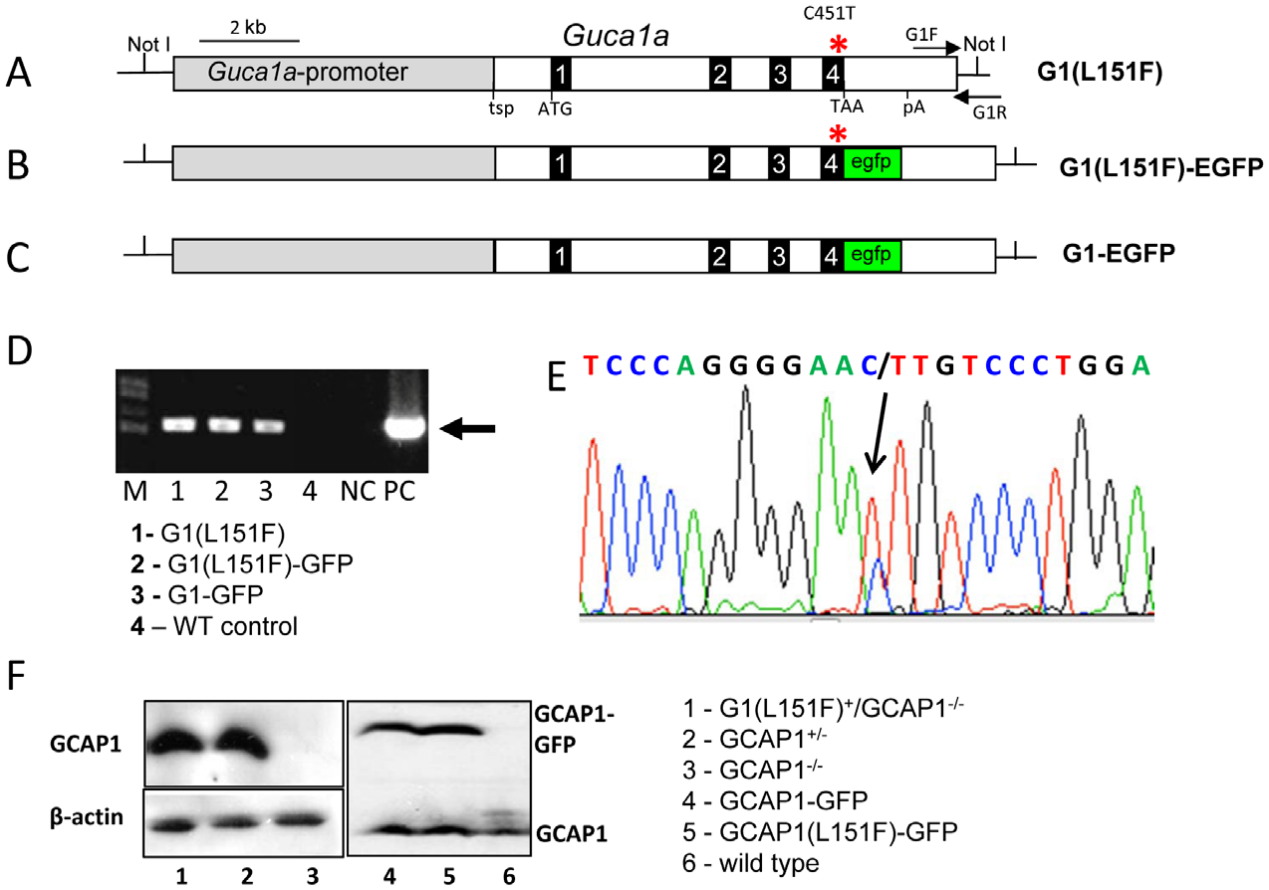
Generation of transgenic mouse lines, GCAP1-EGFP and GCAP1(L151F). Schematic of three mouse GCAP1 genomic transgene constructs: G1(L151F) (**A**), G1(L151F)-GFP (**B**) and G1-GFP (**C**). Black boxes depict exons 1–4. Tsp, transcription start point; ATG and TAA, translation start and stop codons; pA, polyadenylation signal. All transgenes contain the entire GCAP1 genomic sequence, including 6.2 kb promoter and 2.5 kb 3′-UTR sequence. Oligonucleotide pair (horizontal arrows, **A**), G1F and G1R, were used for genotyping. The C451T point mutation was introduced at exon 4 (red stars) resulting in a L151F mutation of G1(L151F) and G1(L151F)-GFP transgenes. In transgenes G1(L151F)-GFP and G1-GFP, EGFP was inserted immediately upstream of the stop codon. **D.** Genotyping of wildtype and three GCAP1 transgenic mice by PCR-amplification. The 540-bp DNA fragment amplified from tail DNA of mouse lines A–C, but not from a wildtype control (mouse 4). Wildtype mouse DNA and transgene plasmids were used as negative (NC) and positive controls (PC), respectively. M, 1 Kb plus DNA ladder; **E.** Representative DNA sequence showing the point mutation C451T (black arrow) in transgenic mice lines A and B. **F.** Immunoblot analysis of transgenic GCAP1 expression using anti-GCAP1 antibody, UW 101. The source of each retina lysate and its lane (1–6) on the blot is indicated (right). β-actin (lanes 1–3) and endogenous GCAP1 (lanes 4–6) are loading controls.

We used the mouse *Guca1a* gene as a foundation to construct three transgenes. The L151F mutation was introduced in exon 4 to establish two GCAP1 transgenic mouse lines, G1(L151F) (**Fig. 2A**) and G1(L151F)-GFP (**Fig. 2B**). The GCAP1-EGFP fusion allowed detection of transgene expression by live fluorescence microscopy, and distinction of transgenic GCAP1 (50 kDa) from native GCAP1 (23 kDa). A third line expressed a GCAP1-EGFP fusion protein containing L151 (no L151 mutation) as a WT control (**Fig. 2C**). The C-terminal in-frame fusion with EGFP was achieved using the homologous recombination method, Red/ET recombination cloning, which is a DNA manipulation technique that is independent of the presence of restriction sites and the size of the DNA molecule to be modified [27] (see Methods). Primers located near the 3′-end of the transgene and in the multiple cloning site of the pBSKS(+) backbone allowed precise genotyping of transgenic mice (**Fig. 2D**). The L151F mutation in G1(L151F) and G1(L151F)-GFP transgenic mice was confirmed by sequencing (**Fig. 2E**).

Relative expression of the GCAP1 transgenes in mouse retinas was examined by immunoblotting retinal lysates at 1–2 months of age (**Fig. 2F**). Antibody directed against mouse GCAP1 (UW101) recognized both endogenous GCAP1 and transgenic GCAP1 fusion proteins. To identify expression of G1(L151F) which co-migrates with native GCAP1, we crossed the G1(L151F) transgenic line with GCAP^−/−^ mice. UW101 detected a 23 kDa polypeptide in G1(L151F)^+^/GCAP^−/−^ retinal lysates (**Fig.** **2F**, lane 1) comparable to the expression level of GCAP1^+/−^ retinal lysate expressing only one allele (**Fig.** **2F**, lane 2). As expected, no GCAP1 was detected in GCAP^−/−^ retinal lysates (**Lane 3**). In G1(L151F)-GFP and G1-GFP transgenic retinas, GCAP1-GFP fusion proteins (∼50 kDa) and native GCAP1 (23 kDa) were co-expressed (**Fig. 2F**, lanes 4 and 5). Immunoblot intensities of the 50 kDa fusion proteins suggest that transgenes G1(L151F)-GFP and G1-GFP are expressed at similar levels.

### Subcellular Distribution of Transgenic GCAP1 Proteins

**Figure 3:**
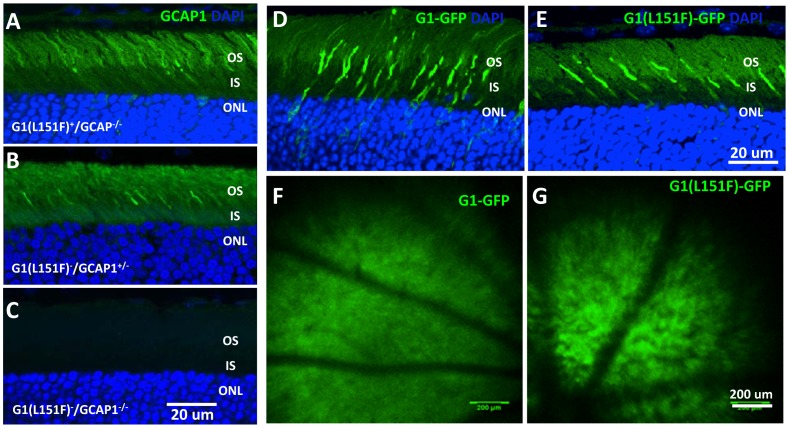
Confocal immunolocalization of transgene products (A-C), direct fluorescence (D,E) and fundoscopy (F,G) of transgenic mouse retinas. Immunohistochemical distribution of G1(L151F)-GCAP1 protein in transgenic mouse retina sections using the anti-GCAP1 antibody, UW101. When the mutant transgene is bred onto a GCAP1/2 double-knockout background, label attributed to mutant GCAP1 (green) is observed in rods and cones of the G1(L151F)^+^/GCAP^−/−^ transgenic retina (A). Comparison to GCAP^+/−^ (heterozygous knockout of endogenous GCAP1, B) retina reveals similar immunolabel intensity, with slight enrichment in cones whereas label is absent in GCAP^−/−^ retina (C). Direct fluorescence microscopy to investigate subcellular localization of G1-GFP and G1(L151F)-GFP transgenes expressed in the mouse retinas. Both G1-GFP (D) and G1(L151F)-GFP (E) transgenes are expressed in photoreceptors, particularly in cones, as shown by GFP fluorescence. F,G. *In-vivo* fluorescence fundus photography visualizing retinal expression of G1-GFP and G1(L151F)-GFP in the transgenic mice. Abbreviations: OS, outer segment; IS, inner segment; ONL, outer nuclear layer stained by DAPI, 4′,6-diamidino-2-phenylindole, a nuclear marker (blue).

We next analyzed the subcellular distribution of G1(L151F) by immunohistochemistry and confocal microscopy. When expressed on the GCAP1/GCAP2 null background, GCAP1(L151F) localized to the photoreceptor inner and outer segments and exhibited a stronger signal in cones than in rods (**Fig. 3A**), comparable to endogenous GCAP1 (**Fig. 3B**) [5]. As predicted, the GCAP double knockout mouse retina was negative for GCAP1 immunolabeling (**Fig. 3C**). Direct fluorescence microscopy of retinal cryosections of G1-GFP and G1(L151F)-GFP transgenic mice, contrasted with diamidino-2-phenylindole (DAPI) nuclear stain, revealed that both G1(L151F)-EGFP and G1-EGFP transgene products were expressed in the photoreceptor inner and outer segments (**Fig. 3D, E**). Fluorescence attributable to transgenic wildtype and mutant GCAP1s was observed in photoreceptors residing in the most sclerad three layers of the ONL, i.e., cones, where the perinuclear accumulation is most likely caused by ‘overexpression’ when the transgene product exists on a background of endogenous (two alleles) GCAP1. Expression of G1(L151F)-GFP and G1-EGFP in transgenic mouse retinas could be discerned easily by *in-vivo* fluorescence (**Fig. 3F, G**).

### Slowly Progressive Photoreceptor Degeneration in GCAP1 Mutant Transgenic Mice

**Figure 4:**
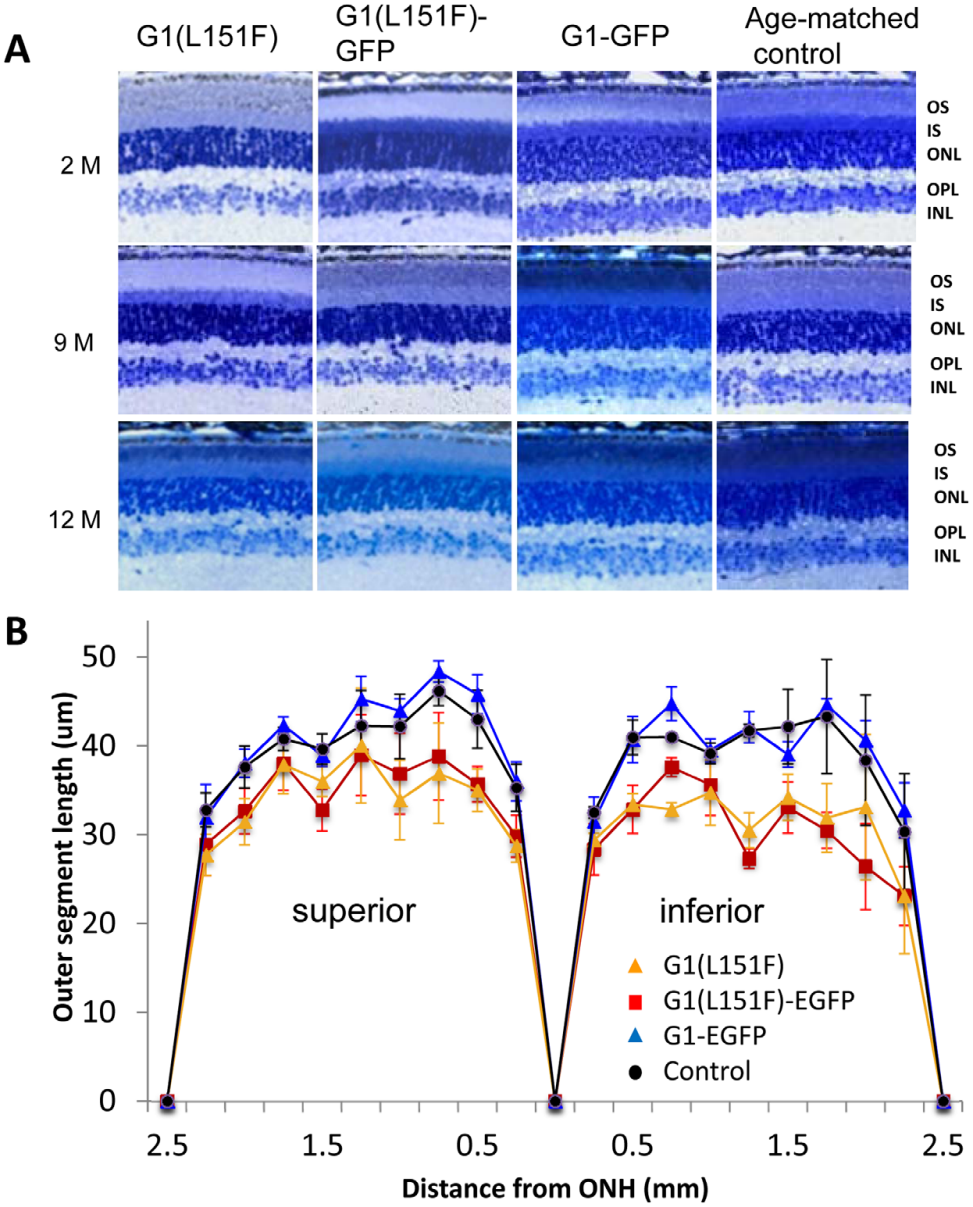
Expression of mutant GCAP1(L151F) results in slowly progressive photoreceptor degeneration. Retinal morphology of the three GCAP1 transgenic lines and age-matched controls were analyzed with plastic retinal sections at ages 2, 9 and 12 months (**A**). Retinas from G1 (L151F) and G1(L151F)-GFP mutant transgenic mice show no morphological change at age 2 months (top, left two panels) compared to control retinas (top, right two panels). At 9 months of age, a thinned photoreceptor layer (including OS, IS and ONL) becomes evident with ∼10% reduction (middle, left two panels). Photoreceptor layer thinning became more obvious at 12 months with ∼20–30% reduction (bottom, left two panels). In contrast, G1-GFP and age-matched wildtype photoreceptor layers are normal at all three ages (bottom, right two panels). Abbreviations: OS, outer segment; IS, inner segment; ONL, outer nuclear layer; OPL, outer plexiform layer; INL, the inner nuclear layer. **B.** Quantitation of the photoreceptor outer nuclear layer (ONL) thickness in three GCAP1 transgenic mice and age-matched controls at 12 months of age. ONL thickness was measured every 250 µm moving from the optic nerve head (ONH) to the periphery, both inferiorly and superiorly. ONL thickness was reduced in retinas of G1(L151F) and G1(L151F)-GFP transgenic mice, but not in G1-GFP transgenic or wildtype mice. (n = 3 each group).

The onset of retinal degeneration was determined by histology, electroretinography and behavioral testing of live animals. Transgenic G1(L151F), G1-GFP and age-matched wildtype mouse retinas were indistinguishable early (**Fig. 4A**, top panels at 2 months). Further, all three GCAP1 transgenic lines showed normal scotopic and photopic ERG responses at two months (data not shown). However, slight reduction of scotopic (a-wave) and photopic (b-wave) ERG amplitudes in 9 month-old mutant transgenic mice signaled the onset of photoreceptor degeneration, and this was reflected by a modest thinning of the ONL (**Fig. 4A**, middle panels). Degeneration in both mutant mouse models progressed slowly, revealing ∼10–20% reduction of ONL thickness compared to wildtype controls and was particularly obvious in the inferior retina (**Fig. 4A**, bottom panels at 12 months; **Fig. 4B** **,** **Table 1**).

**Table 1:** Outer nuclear layer (ONL) thickness of GCAP1 transgenic mice and age-matched controls.

	Distance from ONH (mm)	0.25	0.5	0.75	1	1.25	1.5	1.75	2	2.25	2.5
**1**	G1(L151F)	29.4±0.8	33.4±1.2	32.8±0.8	34.7±3.7	30.5±2.0	34.2±2.6	31.9±3.9	33.1±8.2	23.1±6.5	0
**2**	G1(L151F)-GFP	28.3±2.9	32.8±2.7	37.6±1.1	35.6±3.5	27.3±1.1	33.1±2.9	30.5±2.0	26.4±4.8	23.1±3.3	0
**3**	G1-GFP	31.5±2.7	40.7±2.6	44.7±1.9	39.5±1.3	42.1±1.8	39.0±1.4	44.6±0.7	40.6±2.2	32.8±3.1	0
**4**	Age-matched Control	32.5±0.6	41.0±1.9	41.0±0.4	39.2±1.2	41.7±0.7	42.2±4.2	43.3±6.4	38.4±7.3	30.4±6.5	0
	Distance from ONH (mm)	0.25	0.5	0.75	1	1.25	1.5	1.75	2	2.25	2.5
**5**	G1(L151F)	28.8±1.8	35.0±2.4	36.9±5.7	33.9±4.5	40.0±6.5	36.0±1.7	37.9±3.3	31.4±2.6	27.7±2.3	0
**6**	G1(L151F)-GFP	29.8±2.4	35.7±2.0	38.8±4.9	36.9±4.6	39.0±4.6	32.8±2.4	38.0±3.0	32.7±2.6	28.9±3.5	0
**7**	G1-GFP	36.0±2.2	45.8±2.2	48.4±1.2	44.0±1.3	45.3±2.6	38.9±1.2	42.3±1.0	38.1±1.6	32.0±3.7	0
**8**	Age-matched Ctr	35.3±2.7	43.0±3.3	46.2±1.7	42.2±3.6	42.7±4.0	39.7±1.7	40.8±1.3	37.6±2.3	32.8±1.9	0
**9**	G1(L151F) vs Ctr					p<0.001*					
**10**	G1(L151F)-GFP vs Ctr					p<0.001*					
**11**	G1(L151F)-GFP vs G1-GFP					p<0.001*					
**12**	G1-GFP vs Ctr					p = 0.09					

Rows 1–4, distances from the optic nerve head (ONH) in inferior hemispheres; rows 5–8, distances from ONH in inferior hemispheres at 0.25 mm intervals. Rows 9–10, p values of distance comparison between different groups.

* p<0.05.

**Figure 5:**
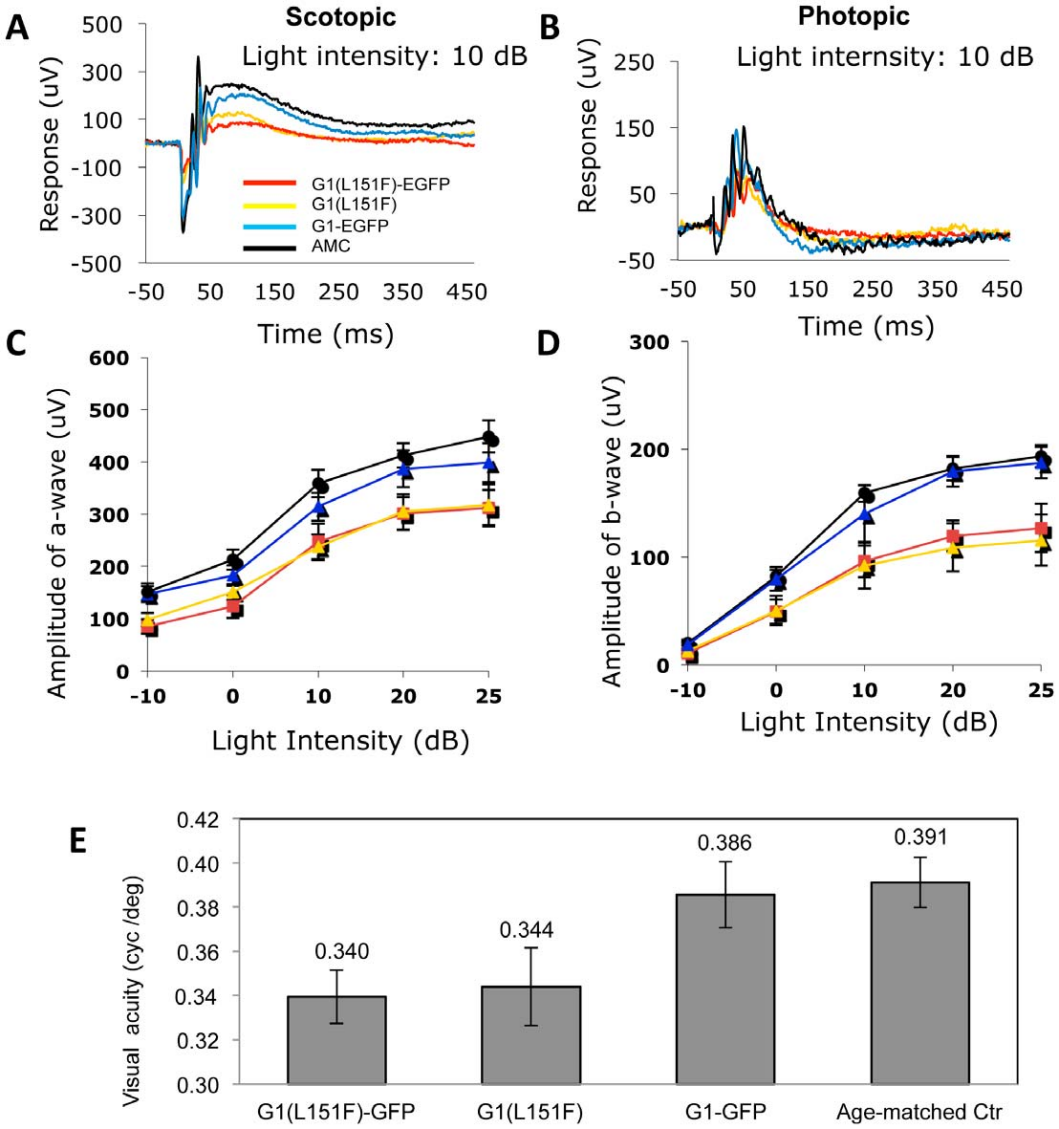
Retinal function of three GCAP1 transgenic mouse lines evaluated by ERG and Optomotry. Scotopic (**A**) and photopic (**B**) ERG analysis at age 12 months (n = 5 mice per genotype). Average a-wave amplitudes of the scotopic responses (**C**) and b-wave amplitudes of the photopic responses (**D**) as a function light intensity. Error bars are standard deviations. **E.** Visual acuities of GCAP1 transgenic mice versus age-matched controls (n = 4) were tested by Optomotry at 12 months of age. Error bars are standard deviations, *p<0.05.

Attenuated scotopic and photopic ERG responses became significant at 12 months, especially under high input light intensities. For example, under a 10 dB flash intensity, the mean scotopic a-wave amplitudes measured in G1(L151F) and G1(L151F)-GFP mice were 238 µV(±23 µV, n = 5) and 247 µV(±35 µV, n = 5). These values are reduced by 20–30% relative to age-matched wildtype control (358±26 µV, n = 5) and G1-GFP transgenic mice (314±27 µV, n = 5) (**Fig. 5**, **Table 2**). The mean photopic b-wave amplitudes measured in G1(L151F) and G1(L151F)-GFP transgenic mice, 92 µV (±22 µV, n = 5) and 96 µV (±15 µV, n = 5) respectively, were reduced by 30–40% relative to age-matched wildtype (159±8 µV, n = 5) and G1-GFP (140±26 µV, n = 5) transgenic mice.

**Table 2:** ERG a- and b- wave amplitudes recorded from wildtype and GCAP1 transgenic mice.

	Light intensity (dB)	−10	0	10	20	25
		a-wave amplitude of scotopic ERG response (µV)				
1	G1(L151F)	95±13	141±11	226±9	285±28	296±34
2	G1(L151F)_GFP	80±11	117±21	240±40	294±35	311±43
3	G1_GFP	147±15	209±17	347±27	403±30	428±31
4	Age-matched Ctr	141±16	196±9	338±17	401±20	436±39
5	G1(L151F) vs Ctr			p<0.001*		
6	G1(L151F)_GFP vs Ctr			p<0.001*		
7	G1(L151F)_GFP vs G1_GFP			p<0.001*		
8	G1_GFP vs Ctr			p = 0.61		
		b-wave amplitude of photopic ERG response (µV)				
9	G1(L151F)	13±4	46±7	95±22	108±17	113±19
10	G1(L151F)_GFP	10±2	45±11	90±11	115±14	121±23
11	G1_GFP	20±5	90±7	154±8	183±14	184±10
12	Age-matched Ctr	19±2	83±3	163±9	185±13	199±12
13	G1(L151F) vs Ctr			p<0.001*		
14	G1(L151F)_GFP vs Ctr			p<0.001*		
15	G1(L151F)_GFP vs G1_GFP			p<0.001*		
16	G1_GFP vs Ctr			p = 0.25		

Rows 1–4, a-wave amplitudes of scotopic ERG responses (µV). Rows 5–8, p values of a-wave amplitude comparison between different groups, Rows 9–12, b-wave amplitudes of photopic ERG responses (µV), Rows 13–16, p values of b-wave amplitude comparison between different groups.

* p<0.05.

Correspondingly, Optomotry at 12 months of age showed marked visual impairment in mice expressing the mutant GCAP1 transgenes. Cone-mediated behavior of G1(L151F) and G1(L151F)-GFP transgenic mice showed threshold reduction of ∼10–15% compared to that of age-matched controls and G1-GFP transgenic mice (**Fig. 5E**). Thus, no significant difference between G1-GFP transgenic and age-matched wildtype mice were observed with regard to morphology (**Fig. 4**), scotopic a-wave (p = 0.085) and photopic b-wave amplitudes (p = 0.074) (**Fig. 5A–D** **,** **Table 1**) or visually guided behavior (**Fig. 5E**). These tests reveal that the GCAP1(L151F) mutation causes a slowly progressing cone-rod dystrophy, first recognizable at around 9 months, progressing to a 20% reduction in ONL thickness, 40% reduction in cone b-wave amplitude, and 15% reduction in visual acuity by one year of age.

### Identification of Anti-mGCAP1 shRNAs *in vitro*

**Figure 6:**
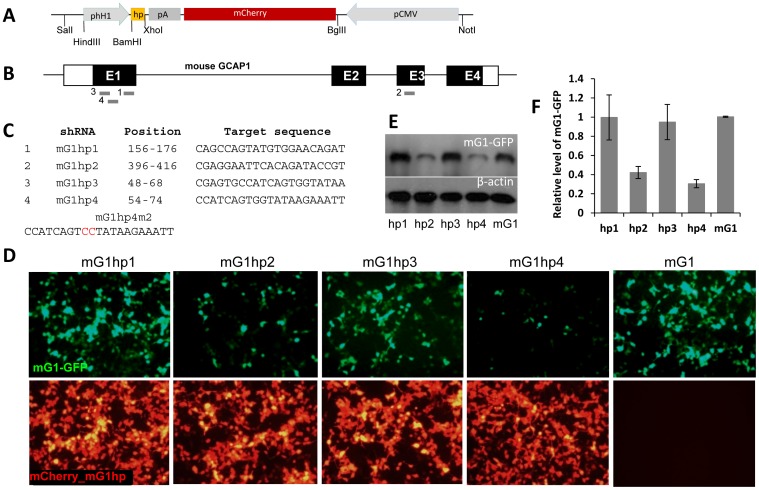
Knockdown of mouse GCAP1 *in vitro* by shRNAs. Schematic (**A**) shows shRNA expression construct that contains a 68-bp shRNA cassette driven by the human H1 promoter, and a CMV promoter-driven mCherry reporter gene. **B.** Schematic of the mouse GCAP1 gene and four candidate anti-mGCAP1 shRNAs, mG1hp1-4, each targeting a different exomic location. **C.** Targeting sequences of mG1hp1-4 and their positions within the GCAP1 gene. mG1hp4m2 is a mismatched control of mG1hp4 in which the central GG was replaced by CC (red). **D.** Knockdown efficiencies of anti-mGCAP1 shRNAs as determined by fluorescence microscopy of HEK293 cells 48 hr after cotransfection with mGCAP1-GFP and each anti-mGCAP1 shRNA, mG1hp1-4. Top row, GFP signal is diminished in cultures transfected with mG1hp2 and mG1hp4, indicating knockdown of mGCAP1 protein. Bottom row, mCherry reporter co-expression of mG1hp1-4 reveals comparable transfection efficiencies among the cultures. In the bottom far right panel, culture “mG1” was transfected with mGCAP1-GFP plasmids only as a non-knockdown control. **E.** Western blot analysis of transfected cell lysates at 50 hr post-transfection. mGCAP1-GFP was detected by anti-GCAP1 antibody, UW101, with β-actin immunostaining as the endogenous loading control. **F.** Relative levels of mGCAP1-GFP expression in the 50 hr transfected cultures were quantified by immunoblot band intensities and normalized against β-actin. The relative level of mG1-GFP in each cotransfected cell sample was averaged from three tests, representing the knockdown efficiency of each shRNA at different concentrations. Knockdown was most pronounced by transfection with mG1hp4, showing ∼70% decrease compared to mG1 non-knockdown control.

Germline deletion of GCAPs in mouse display neither gross functional deficiencies nor retinal degeneration [8], [9]. A nonallele-specific knockdown of *Guca1a* mRNA in the G1(L151F) transgenic mice may therefore be a promising tool for RNAi- based gene therapy to cure dominant CORD caused by GCAP1 mutations. Using online shRNA prediction tools, we designed four anti-mGCAP1 candidate shRNAs which have no significant homology with other mouse genes. To identify an effective anti-mGCAP1 shRNA, we developed an *in-vitro* shRNA screening system in which disappearance of mGCAP1-EGFP fluorescence would be proportional to the knockdown efficiencies of shRNA candidates [4]. We used a shRNA expression construct with a mCherry reporter (**Fig. 6A**) to test four anti-mGCAP1 shRNA candidates, mG1hp1-4 (**Fig. 6B, C**), in which shRNA transcription is controlled by the human H1 promoter. Target mG1-EGFP plasmids were co-transfected into HEK293 cells with each anti-mGCAP1shRNA plasmid at a 1∶3 mass ratio. Live cell imaging revealed that mG1hp2 and 4 significantly suppressed mG1-EGFP expression in the co-transfected cells whereas mG1hp1 or 3 were largely ineffective (**Fig. 6D**). Western blotting of the cell lysates verified that mG1hp2 and 4 knocked down mG1-EGFP with 60% and 70% efficiency, respectively (**Fig. 6E, F**).

**Figure 7:**
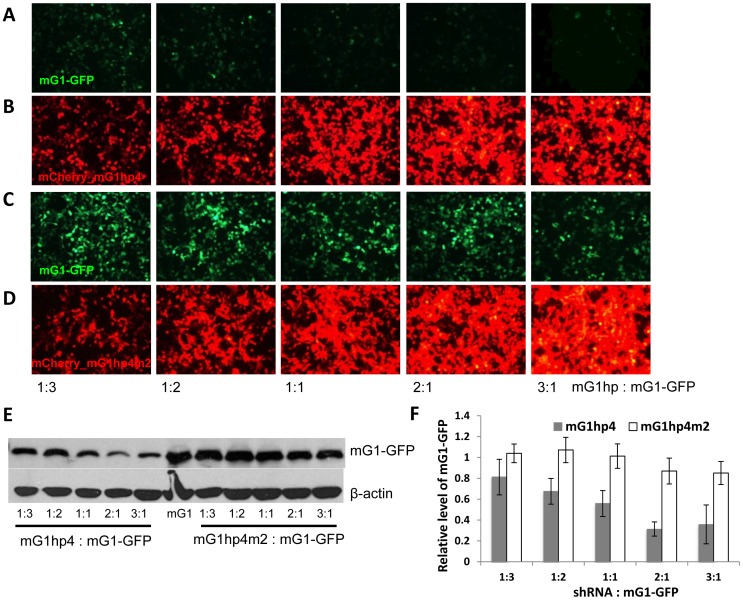
Dose-dependent and sequence-specific knockdown of mGCAP1 by mG1hp4. A–D. Fluorescence microscopy of HEK293 cells 48 hrs after cotransfection with mGCAP1-EGFP and mCherry_mG1hp4 (**A, B**) or its mismatched control mCherry_mG1hp4m2 (**C, D**) at five different mass ratios. GFP signal in row A is gradually diminished as mG1hp4 increases to a saturation level, indicating knockdown dose-dependency from 1∶3 to 2∶1. In row C there is no obvious change of GFP signal in cultures from 1∶3 to 2∶1 since the mutant mCherry_mG1hp4m2 is inactive for suppression of mGCAP1. Panels of rows B and D show increase in mCherry signal corresponding to increased mass ratios, left to right. **E.** Representative western blot evaluating mGCAP1-GFP protein levels in the cotransfected cells (50-hr post-transfection); mGCAP1-GFP was detected by anti-GCAP1 antibody, UW101, with β-actin immunostaining as the loading control. **F.** Quantification of relative mGCAP1-GFP levels shown in (**E**). The intensities of the mG1-GFP specific bands in western blot were measured by using ImageJ, and normalized against β-actin. The relative level of mG1-GFP in each cotransfected cell sample was averaged from three tests, representing the knockdown efficiency of each shRNA at different concentrations.

To determine knockdown specificity of mG1hp4, a 2-nucleotide mismatch control, mG1hp4m2was generated by mutating the central two nucleotides in the mG1hp4 guide strand (GG to CC) (**Fig. 6C**). We then cotransfected mG1-GFP and mG1hp4/mG1hp4m2 plasmids into HEK293 cells at five different mass ratios (1∶3, 1∶2, 1∶1, 2∶1, and 3∶1). Fluorescence live cell imaging of cotransfected cells 48 hr post-transfection showed that mG1-GFP levels were incrementally reduced with increasing amounts of mG1hp4 from 1∶3 to 2∶1, as evidenced by mCherry fluorescence (**Fig. 7A, B**). No significant suppression of mG1-EGFP was observed when mG1hp4 was replaced by mG1hp4m2 at the mass ratio of mG1hp4m2/mG1-GFPfrom 1∶3 to 2∶1 (**Fig. 7C, D**). However, when shRNA increased to the mass ratio of 3∶1, knockdown efficiency of mG1hp4 did not rise further, and mG1hp4m2 presented a nonspecific suppression effect on mG1-EGFP. Western blot analysis of the cell lysates at 50 hr post-cotransfection confirmed that mG1-GFP was reduced from 70% to 30% as shRNA increased from 1∶3 to 2∶1, whereas mutant shRNA showed ∼15% reduction at 2∶1 and 3∶1 (**Fig. 7E, F**). These results demonstrate that mG1hp4 could efficiently and specifically suppress mG1-GFP expression. A nonspecific effect was observed in mG1hp4m2 at very high shRNA/mGCAP1-EGFP ratios.

### Visualization of shRNA-mediated Gene Silencing *in-vivo*

**Figure 8:**
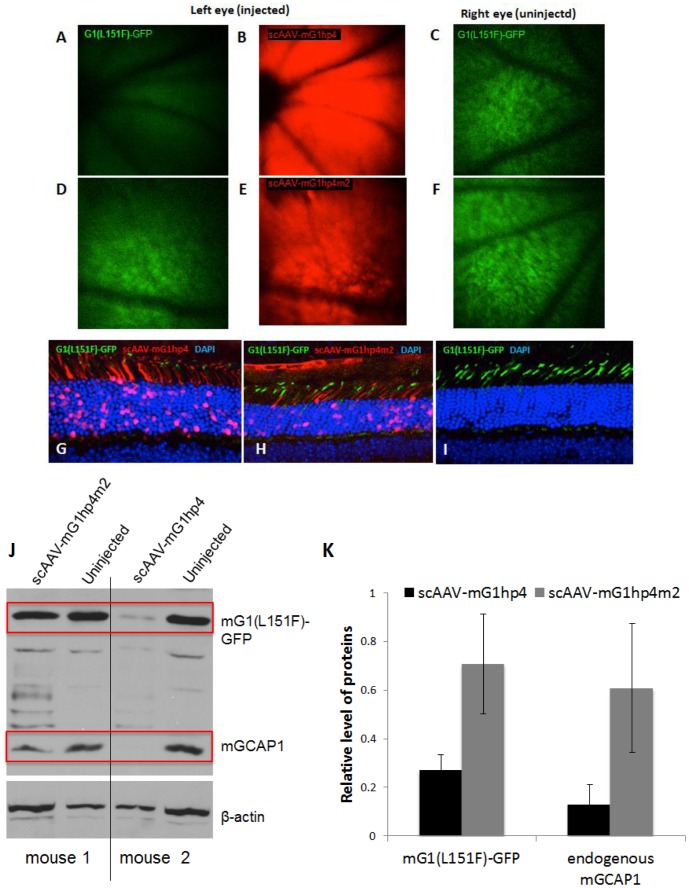
*In-vivo* knockdown of transgenic mGCAP1 by therapeutic AAV2/8-mG1hp4. A–C. Representative fluorescence fundus images of G1(L151F)-GFP transgenic mice with subretinal injection of scAAV2/8-mG1hp4 to the left eyes. GFP signal representing the G1(L151F)-GFP level in photoreceptors is diminished in the left eye at 30 days post-injection (**A**), compared to that in the uninjected right eye (**C**), indicating significant knockdown of G1(L151F)-GFP by mG1hp4 whose expression is shown by mCherry reporter signal (**B**). **D–F.** Representative fluorescence fundus images of G1(L151F)-GFP transgenic mice with subretinal injection of scAAV2/8-mG1hp4m2 (2-bp mismatch control) in their left eyes. In contrast to scAAV2/8-mG1hp4-treated eyes (**A**), there is no obvious knockdown of G1(L151F)-GFP by scAAV2/8-mG1hp4m2 (**D**), compared to the uninjected right eye (**F**). Even mG1hp4m2 is expressed at levels comparable to mG1hp4 in the treated eye, as shown by mCherry reporter (**B, E**). **G–I.** Fluorescence microscopy of retinal sections of G1(L151F)-GFP transgenic mice receiving scAAV2/8-mG1hp4 or -hp4m2 in their left eyes. Compared to uninjected right eyes (**I**), G1(L151F)-GFP signal in the outer nuclear layer decreased significantly with mGhp4 expression, but less with mG1hp4m2 expression. **J.** Immunoblot analysis of G1(L151F)-GFP transgenic mouse retinas 30 days after subretinal injection. Transgenic G1(L151F)-GFP and endogenous GCAP1 proteins were detected by anti-GCAP1 antibody, UW101. β-actin served as endogenous loading control. Compared to the uninjected right retinas (lanes 2 and 4), both G1(L151F)-GFP (∼50 kD) and endogenous GCAP1 (∼25 kD) are decreased in the left retina treated with AAV-mG1hp4 (lane 3), but not when treated with AAV-mG1hp4m2 (lane 1). **K.** Quantification of relative G1(L151F)-GFP and mGCAP1 levels in retinas 30 days post-injection with AAV8-mG1hp4 and AAV8-mG1hp4m2. G1(L151F)-GFP and mGCAP1 levels were averaged from 3 retinas of each vector treatment, and normalized against β-actin. Relative to uninjected eyes, G1(L151F)-GFP and mGCAP1 were knocked down ∼75% and ∼90% by AAV-mG1hp4, and 30% and 35% by AAV-mG1hp4m2.

We packaged mG1hp4 and mG1hp4m2 into scAAV2/8 vectors that express shRNA rapidly and persistently in mouse photoreceptors [4]. The scAAV8-mG1hp4 virus was injected subretinally into the left eyes of G1(L151F)-GFP mice at 1 month of age, before appreciable photoreceptor degeneration has occurred. At one week post-injection, mice exhibiting mCherry expression in >50% of the retinal area (visualized by live fluorescence imaging) were retained for further analysis. Fluorescence fundus photographs of both treated and untreated eyes were taken from the G1(L151F)-GFP transgenic mice one month after the injection. In treated left eyes, the extent of mCherry expression covered nearly the whole retina suggesting successful and widespread vector-mediated expression (**Fig. 8B**). In those retinas expression of transgenic G1(L151F)-GFP was significantly suppressed (**Fig. 8A**) compared to that seen in untreated right eyes (**Fig. 7C**). In contrast, scAAV8-mG1hp4m2 treatment in the left eyes had no detectable effect (**Fig. 8D–F**).

To verify the fundoscopy, retinal cryosections of both left and right retinas from scAAV8-mG1hp4 or scAAV8-mG1hp4m2-treated mice were analyzed by direct fluorescence microscopy (**Fig.** **8G–I**). G1(L151F)-GFP expression was nearly completely suppressed in the scAAV8-mG1hp4- treated retinas (**Fig. 8G**), but to a much lesser extent in retinas treated with scAAV8-mG1hp4m2 (**Fig. 8H**) consistent with live fundoscopy. Further, expression levels of endogenous GCAP1 in the treated mice were analyzed by immunoblot. In the scAAV-mG1hp4 treated retinas, both transgenic G1(L151F)-GFP and endogenous GCAP1 were significantly suppressed while the mutant shRNA vector had no significant effect (**Fig. 8J**). Quantitative analysis of GCAP1 levels (n = 3) revealed ∼70% knockdown of G1(L151F)-GFP transgene, and 90% knockdown of endogenous GCAP1 in the scAAV-mG1hp4-treated G1(L151F)-GFP retinas, but only 30–35% knockdown in the scAAV-mG1hp4m2-treated retinas, which probably was caused by nonspecific effects at a high shRNA concentration (**Fig. 8K**).

## Discussion

The purpose of this study was to generate mouse models of dominant CORD based on a GCAP1(L151F) mutation associated with human disease. The mouse models serve as tools to explore the efficiency of a nonallele-specific knockdown strategy suitable for human gene therapy. The transgenes consisted of a 15 kb *Guca1a* genomic fragment containing the native regulatory elements including endogenous promoter, all introns and endogenous polyadenylation sites. In the GCAP1-EGFP and GCAP1(L151)-EGFP fusion models, the coding region of exon 4 of the GCAP1 gene was fused to EGFP cDNA (**Fig. 2B, C**). Fusion of EGFP to GCAP1 at its C-terminus provided means to directly visualize GCAP1 localization by live fluorescence retina imaging (**Fig. 3**) and to monitor the suppression of GCAP1 by RNA interference (**Fig. 8**). The transgenes expressed wildtype and mutant GCAP1s in mouse photoreceptors at a level similar to endogenous GCAP1 in heterozygous mice (GCAP^+/−^). No ectopic transgene expression was observed apart from accumulation of transgenic GCAP1 in perinuclear locales of a few individual cones (**Fig. 3D**).

In human patients carrying GCAP1 mutations, phenotypes are noticed typically within the second or third decade and progress slowly [2]. In transgenic mice expressing GCAP1(L151F), we observed reduced thickness of the photoreceptor layer as early as 9 months of age (**Fig. 4**). At 12 months of age, reduction of photopic ERG b-wave amplitudes was more pronounced than that of scotopic a-waves suggesting that cones degenerate somewhat faster than rods in these mouse models (**Fig. 5**). As mouse cones express GCAP1 only, a more severe L151F dominant negative effect is expected in these cells. Another, more severe, EF4-hand mutation associated with CORD (E155G) causes reductions in cone function in patients starting at an average age of 16 years, about 10 years earlier than for L151F patients [16], [17], [23]. Consistent with this more aggressive degenerative human phenotype, far greater functional deficits in cones than rods were observed in the E155G knock-in mouse model [26]. Starting at 3 months postnatally, photopic ERG responses of E155G knock-in mice were reduced and the flicker response was severely depressed. At 12 months, the photopic ERG b-wave was reduced to 42% of normal, compared to 60% in our L151F transgenic mice. The presence of two normal *Guca1a* alleles in our L151F model versus just one in the E155G knock-in model may account for the phenotype differences. Further, the E155G mutation in which an essential charged residue is replaced by a neutral one represents a much more severe EF-hand structural change than seen in L151F. Nevertheless, our ‘genomic transgenic’ mouse yields phenotypes comparable to the ‘knock-in’ mouse suggesting that the 15 kb genomic fragment harboring Guca1a contains all the essential regulatory elements.

Functional differences in the transgenic mice at one year of age relative to age-matched wildtype mice are relatively mild (**Figs. 4**, **5**), similar to those of second and third decade GCAP1(L151F) patients. The GCAP1(L151F) mutation was identified in two unrelated, five-generation families affected with cone and cone-rod dystrophy [17], [23]. In young patients, rod and cone ERGs were near normal. Loss of cone function occurs first and rod function generally persists much longer in CORD families. Although the disease phenotypes were variable among the affected members in both families, most patients experienced late-onset cone dysfunction by the second and third decade of life. Another important CORD mutation is N104K in EF-hand 3 which we investigated previously [20]. A proband carrying a GCAP1(N104K) mutation and diagnosed by fundoscopy with dominant CORD at 39 years, experienced a slow loss of acuity over the course of 12 years [20]. Considering differences in human and mouse lifetimes, our late-onset and slowly progressive transgenic mouse models reveal very similar features and compare well with the human disease. The subtle deviations may be explained by the presence of two normal *Guca1a* alleles in our transgenic mice and by differences in human and mouse retinal anatomy, as the mouse has neither macula nor cone-rich central fovea.

The principal goal of this research is to identify gene therapy vectors that may be used successfully to delay the onset of cone degeneration, and/or cure CORD disease. In an earlier study, we demonstrated the feasibility of shRNA knockdown using an allele-specific approach in a retinitis pigmentosa mouse model carrying the GCAP1(Y99C) mutation [4]. In that mouse line, a GCAP1(Y99C) cDNA was expressed under the control of the mouse opsin promoter producing a retinitis pigmentosa like phenotype [25]. A therapeutic recombinant AAV robustly and persistently expressed shRNAs in photoreceptors at one week post-injection and silenced the disease-causing bovine GCAP1(Y99C) transgene with ∼80% efficiency; the gene silencing was effective for nearly one year without apparent off-target interference and significantly improved rod photoreceptor survival, delayed disease onset and increased visual function [4].

Germline deletion of GCAPs in mouse delays recovery to the dark state by seconds as guanylate cyclase activity is not accelerated in low Ca^2+^ [8]. However, there are no detrimental morphological consequences, nor have gross functional deficiencies or retinal degeneration been reported. Patients with GCAP1 null or GCAP1/GCAP2 null mutations have not been identified to date, perhaps because a clear degeneration phenotype is absent [30]. We therefore reasoned that a nonallele-specific shRNA knockdown of both wildtype and mutant GCAP1s could be a general therapeutic strategy to rescue the dominant degeneration caused by any of the eight known EF-hand GCAP1 mutations. The most effective anti-mGCAP1 shRNA (mG1hp4) which degraded more than 70% of mGCAP1-EGFP was identified by an *in-vitro* fluorescent screening assay (**Fig. 6**). The “negative control” shRNA that carried a GG to CC mutation in the center was inactive (**Fig. 7**). After packaging into scAAV2/8vector, the mG1hp4 proved to be a potent GCAP1 knockdown agent *in-vivo* as well, as shown by live fluorescence imaging (**Fig. 8**). One month post-injection with AAV8-mG1hp4, we observed significant suppression of GCAP1(L151F)-GFP by 70% in the treated eyes. As expected, knockdown of mutant GCAP1 occurred concomitantly with knockdown of endogenous GCAP1 (∼90%) (**Fig. 8J, K**). As mG1hp4 was designed to specifically target mouse GCAP1 without a C-terminal GFP, AAV-mG1hp4 has shown more efficient suppression of endogenous GCAP1 gene than the GCAP1(L151F)-GFP transgene. These experiments provide proof of principle that RNAi suppression of both wildtype and mutant GCAP1 may be a potent therapeutic strategy, applicable to all GCAP1 mutations of EF-hands 3 and 4, as long as the guide strand of shRNA is located outside the disease-causing mutations. Notably, when shRNA reaches its highest suppression of the target gene, overexpression does not increase efficiency but generates off-target silencing effects on other genes due to saturation of the intracellular RNAi machinery [**31**]. Thus, identification of a highly efficient shRNA may require its titration to find the lowest level efficacious for RNAi-based gene therapy in humans.

An advantage of dominant GCAP1 mutations is that a nonallelic approach promises to be successful, while mutations in other CORD genes require gene replacement for rescue. Dominant CORD is also associated with missense mutations in ***GUCY2D*** (guanylate cyclase 1) (reviewed in [32], ***CRX*** (cone-rod otx-like photoreceptor homeobox transcription factor) [33]–[35], ***AIPL1*** (arylhydrocarbon-interacting receptor protein-like 1) [36]–[38], and ***PROM1*** (Prominin 1) [39], [40]. Proteins encoded by these genes have very diverse functions, yet null mutations of these genes are associated with recessive RP or LCA suggesting expression is vital for photoreceptor survival.
